# Recursive random forest algorithm for constructing multilayered hierarchical gene regulatory networks that govern biological pathways

**DOI:** 10.1371/journal.pone.0171532

**Published:** 2017-02-03

**Authors:** Wenping Deng, Kui Zhang, Victor Busov, Hairong Wei

**Affiliations:** 1 School of Forest Resources and Environmental Science, Michigan Technological University, Houghton, MI, United States of America; 2 Department of Mathematical Sciences Michigan Technological University, Houghton, MI, United States of America; 3 Life Science and Technology Institute, Michigan Technological University, Houghton, Michigan, MI, United States of America; Youngstown State University, UNITED STATES

## Abstract

**Background:**

Present knowledge indicates a multilayered hierarchical gene regulatory network (ML-hGRN) often operates above a biological pathway. Although the ML-hGRN is very important for understanding how a pathway is regulated, there is almost no computational algorithm for directly constructing ML-hGRNs.

**Results:**

A backward elimination random forest (BWERF) algorithm was developed for constructing the ML-hGRN operating above a biological pathway. For each pathway gene, the BWERF used a random forest model to calculate the importance values of all transcription factors (TFs) to this pathway gene recursively with a portion (e.g. 1/10) of least important TFs being excluded in each round of modeling, during which, the importance values of all TFs to the pathway gene were updated and ranked until only one TF was remained in the list. The above procedure, termed BWERF. After that, the importance values of a TF to all pathway genes were aggregated and fitted to a Gaussian mixture model to determine the TF retention for the regulatory layer immediately above the pathway layer. The acquired TFs at the secondary layer were then set to be the new bottom layer to infer the next upper layer, and this process was repeated until a ML-hGRN with the expected layers was obtained.

**Conclusions:**

BWERF improved the accuracy for constructing ML-hGRNs because it used backward elimination to exclude the noise genes, and aggregated the individual importance values for determining the TFs retention. We validated the BWERF by using it for constructing ML-hGRNs operating above mouse pluripotency maintenance pathway and *Arabidopsis* lignocellulosic pathway. Compared to GENIE3, BWERF showed an improvement in recognizing authentic TFs regulating a pathway. Compared to the bottom-up Gaussian graphical model algorithm we developed for constructing ML-hGRNs, the BWERF can construct ML-hGRNs with significantly reduced edges that enable biologists to choose the implicit edges for experimental validation.

## Introduction

There are at least a few hundred metabolic pathways and a few thousand biological processes known to be present in plants and animals, but unfortunately, our knowledge on how these pathways or biological processes are regulated is very limited. For example, *Arabidopsis thaliana* has 549 metabolic pathways (http://www.arabidopsis.org/) and currently the regulators for only a few pathways are partially known. The regulators for most pathways remain elusive. Present knowledge has shown that many pathways and biological processes are regulated by multi-layered hierarchical gene regulatory networks (ML-hGRNs) [1–9]. In the past decade, advances in microarray and RNA-seq technology have generated an enormous wealth of gene expression data. Therefore, it is imperative to develop the methods for reverse-engineering ML-hGRNs from high-throughput gene expression data. ML-hGRNs enable identification of high hierarchical regulators, middle-level and low-level regulators that indirectly or directly govern pathway or biological process genes at bottom-layer. Studies have shown high hierarchical regulators (HHRs) are global modulators that respond to various cellular signals [10, 11] and environmental cues [3, 12]. The middle-level regulatory genes play the manager-like roles or serve as a hub, through which the commands from high hierarchical regulators at upper layers are synthesized and then passed down to low-level regulators that exert regulation on bottom layer genes [3, 13]. We need to identify the high hierarchical regulators at the top levels because they have more pleiotropic effects and are useful if we intend to engineer multiple pathways. To understand how pathways are regulated, we should reconstruct ML-hGRNs that operate above biological pathways and processes. Such ML-hGRNs can provide not only the hierarchies of regulatory genes but also the connectivity among them, which can significantly increase our understanding of wired regulation exerted on metabolic pathways and biological processes through multiple chains-of-command [14].

Although ML-hGRNs are important, there is a lack of statistical or computational methods for directly constructing ML-hGRNs from high-throughput gene expression data [7]. Currently there are many methods that are available for constructing GRNs [15–20], but these methods are not specifically tailored for constructing ML-hGRNs that mimic the hierarchical regulation [7]. We have recently developed an algorithm called Bottom-up Gaussian graphical model (GGM) algorithm [5, 7] for directly constructing ML-hGRNs from high-throughput gene expression data. Although it can recognize the known positive regulators with high efficiency, it usually builds a large number of regulatory relationships (edges) into ML-hGRNs, resulting in possible false positive relationships (edges) and making it hard to identify implicit “top-down chains-of-commands” for experimental validation. In this paper, we developed an new method, called the backward elimination random forest (BWERF), to construct ML-hGRNs based on the random forest modeling [21], with a purpose to reduce the number of false negative relationships (edges) among genes so that the constructed relationships (edges) can represent the true causal relationships.

GENIE3 is gene network inferring algorithm based on the random forest method, and it is the champion of DREAM4 *in silico* network challenge (http://dreamchallenges.org/project/dream4-in-silico-network-challenge/). There are two major differences between BWERF and GENIE3. First, BWERF applies backward elimination to alleviate the interference of noise variables, and allows the regulatory genes with medium-level regulatory strengths surface out from the noise. Second, BWERF aggregates the individual importance values of a TF to all pathway genes to produce a unified importance value of the TF for the pathway, by which the decision for retention can be made more easily. We evaluated the BWERF with three data sets: (1) a synthetic toys data set; (2) a real data set from mouse, and (3) a real data sets from *Arabidopsis thaliana*. We corroborated the high efficiency and accuracy of BWERF algorithm and manifested its uses for recognition of pathway gene regulators through construing ML-hGRNs.

## Results

### Comparison of variable recognizing capability between BWERF and GENIE3 on a simulated “toys dataset”

In order to show the effect of backward elimination, we tested it with a simulated “toys data set”. This data set simulates the expression values of 1 pathway gene (*y*) and 1006 TFs {*X*_1_, *X*_2_,…,*X*_1006_}, in these 1006 TFs, The first 6 TFs {*X*_1_, *X*_2_,…,*X*_6_} are true TFs that regulate the pathway gene and the other 1000 TFs are noise. The setting is as follows:

- X_*i*_∼N(1, 1) for i = 1,2,3
- X_*i*_∼N(3, 1) for i = 4,5,6
- X_*i*_∼N(0, 1) for i = 7,…,1006
- z∼N(0, 0.1)
- *y* = *X*_1_ + *X*_2_ + *X*_3_ + *X*_4_ + *X*_5_ + *X*_6_ + *z*

In such a setting, the 6 true TFs can be divided into two groups {*X*_1_,*X*_2_,*X*_3_} and {*X*_4_,*X*_5_,*X*_6_}. The TFs in first group have weaker signal than the second group, and are more likely to be inundated by the noise TFs. The sample size is set to be 100.

**Fig 1:**
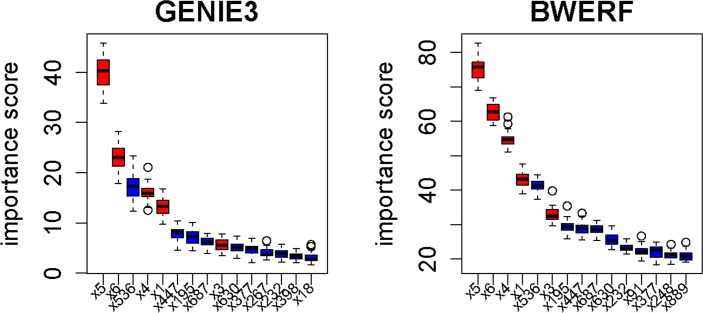
Comparison of the ordered important values of top 15 variables generated by GENIE3 (left) and BWERF (right) on a “toys data set”. Red: true regulators, blue: noise variables. The boxplots were based on 30 runs of GENIE3 and BWERF.

As shown in Fig 1, the strong regulatory TFs *X*_5_ and *X*_6_ were correctly identified as the two most important top TFs by both methods. However, for GENIE3, the noise TF *X*_536_ appeared in the third place, its importance value surpassed the importance values of the other 4 true TFs. In addition, true TF *X*_3_ was inundated by three other noise TFs. When BWERF was applied to this toys data set, the strong regulatory TFs {*X*_4_,*X*_5_,*X*_6_} and weak regulatory TF *X*_1_ had the larger importance values than any noise variables, and true TF *X*_3_ was only surpassed by noise *X*_536_, which clearly manifested the roles of backward elimination in elevating the positions of true positive TFs. We also noticed that the backward elimination implicitly increased the importance values and also their range as backward elimination was advanced, leading to the true regulatory variables becoming more differentiable from noise variables.

### Comparison of ML-hGNRs constructed from mouse gene expression data using BWERF and GENIE3

**Fig 2:**
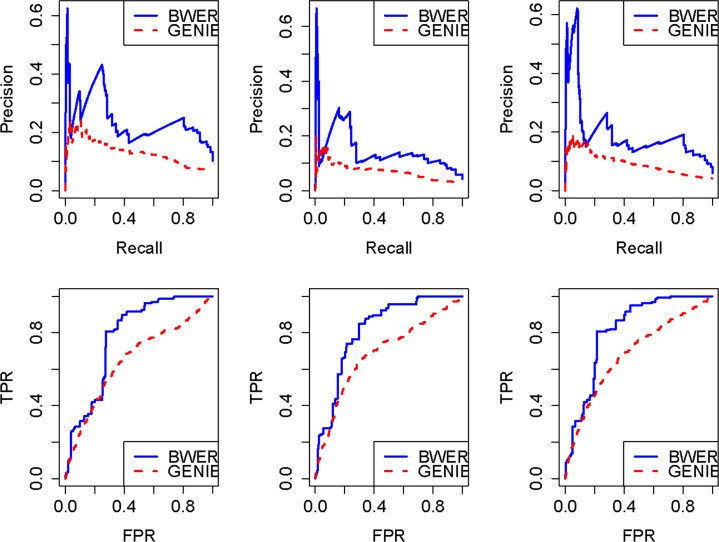
The precision-recall (PR) and receiver operating characteristic (ROC) curves of BWERF and GENIE3 for three mouse microarray data sets. The 24 stem cell pluripotency maintenance genes regulated by the 35 transcription factors (TFs) were obtained from mouse ChIP-seq data. A microarray data set of these pathway genes and TFs yielded from a time course in which the pluripotent cells were subjected to undirected differentiation were downloaded from ESCAPE web portal (http://www.maayanlab.net/ESCAPE/). The three data sets were generated by adding the profiles of 100, 200, and 300 noise variables to the profiles of 35 TF genes for *in silico* experimental validation.

In order to evaluate the performance of BWERF in recognizing regulatory relationships, we downloaded mouse time course gene expression data and ChIP-seq data from embryonic stem cells Atlas of Pluripotency Evidence (ESCAPE). Mouse pluripotency maintenance pathway was selected for demonstrating the effect of BWERF. The 24 genes involved in pluripotency maintenance renewal were chosen for this *in silico* validation. These 24 genes are regulated by 35 known TFs based on the ChIP-seq data provided by ESCAPE web portal. Three test datasets were subsequently generated by adding the profiles of 100, 200, and 300 simulated noise genes into the profiles of the 35 TFs. Since these 35 TFs are the direct regulators of the pathway genes based on ChIP-seq data, we just built one-layered GRN using BWERF and GENIE3. The input data files and the output list of edges with the importance values are provided in S1 File. With the known positive edges, we were able to generate precision-recall (PR) curves and receiver operating characteristic (ROC) curves, and then used them to evaluate the efficiencies of BWERF and GENIE3 (Fig 2). A PR curve plots the proportion of true positives among all predictions versus the percentage of true positives that are retrieved (recall) for varying thresholds on the importance scores while a ROC curve plots the true positive rate versus the false positive rate. The results shown in Fig 2 suggest BWERF had a persistent advantage over GENIE3 in constructing gene regulatory network for a pathway. To summarize these curves, two statistics, the area under PR curve (AUPR) and the area under ROC curve (AUROC) were computed (Table 1). The improvement of BWERF over GENIS was obvious.

**Table 1:** AUPR and AUROC values for mouse datasets using BWERF and GENIE3.

	BWERF			GENIE3		
	Dataset 1	Dataset 2	Dataset 3	Dataset 1	Dataset 2	Dataset 3
PR	0.2405	0.1457	0.1958	0.1312	0.0705	0.1958
ROC	0.7778	0.8150	0.8134	0.6499	0.6868	0.6820

### Comparison of ML-hGNRs constructed from *Arabidopsis* microarray data using BWERF and GENIE3

**Fig 3:**
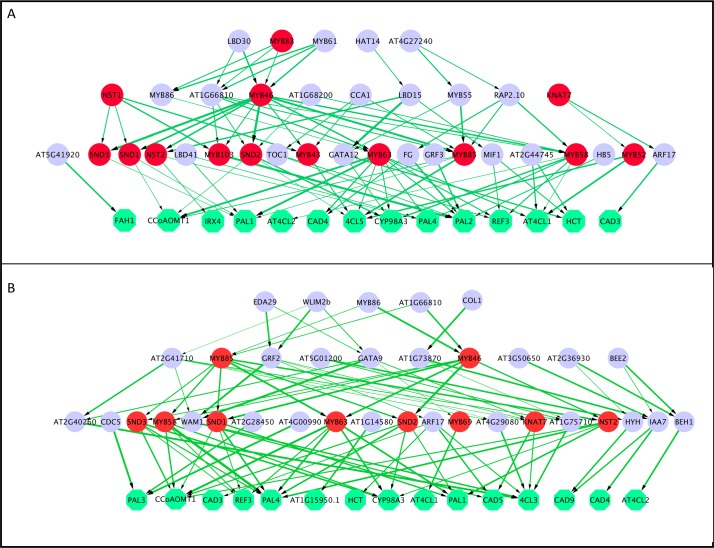
Construction of ML-hGRN for lignocellulosic pathway with a compendium microarray data set (128 chips) from *Arabidopsis thaliana* roots under salt stress condition. A. The four-layered hGRN constructed with the BWERF algorithm. B. The four-layered hGRN built from GENIE3. The input files for BWERF and GENIE3 included the expression profiles of 1602 transcription factors and 22 lignocellulosic pathway genes (green nodes at bottom layers). The nodes with red color highlighted in both networks are known regulatory TFs regulating lignocellulosic pathway in existing knowledgebase. The data, edge list as well as gene IDs represented by each symbol can be found in S2 File.

We built a four-layered hGRN as shown in Fig 3A with 22 lignocellulosic pathway genes being used as bottom and 1602 TF being used as the input for the upper layers. A total of 14 positive TFs including MYB43, MYB46, MYB52, MYB58, MYB63 MYB83, MYB85, MYB103, SND1, 2, 3 and NST1, 2 and KNAT7 [22, 23] were identified as known positive regulators of lignocellulosic biosynthesis. In a previous work, 20 positive known TFs were identified by bottom-up GGM algorithm when 25 pathway genes and 1622 TFs were used [7]. However, these 20 positive TFs were identify by bottom-up GGM in a total of 1507 edges while the BWERF identified the 14 positive TFs in a total of 90 edges. Of these genes, MYB58, MYB63, SND1, 2, 3, and NST2 were located at the secondary layers in both networks built by Bottom-up GGM and BWERF. In the ML-hGRN constructed with BWERF, the regulatory relationships were sparser and more discernible, and the “top-down chains of commands” are much easier to be identified for experimental validation.

In contrast, GENIE3 identified 10 positive TFs that include SND1, 2, MYB58, NST2, MYB63, MYB69, SND3, MYB46 and MYB85 [22] (Fig 3B) for the same inputs as used by BWERF. GENIE3 failed to identify the MYB43, MYB52, MYB83, MYB103 and NST1 that were identified by BWERF, whereas the BWERF failed to identify the MYB69 that was in the network constructed with GENIE3. This comparison again suggests that BWERF be more efficient than GENIE3.

## Discussion

At the time being, little is known about regulatory layers above the majority of over 500 metabolic and canonical pathways. With the availability of terabyte of gene expression data, it is imperative to develop novel methods to construct hGRNs via reverse-engineering approaches. This is because the new methods provide the means to recognize the novel TFs in layered hGRNs that can greatly facilitate our understanding and decipher how biological pathways are regulated. In our earlier work, a bottom-up GGM algorithm was developed to accomplish this goal through implementation of a hypothesis testing (Wald test-based approach) to each combined triple genes: one TF from TF pool and two current bottom-layered genes, leading to identification of the significant triple regulatory blocks for building a ML-hGRN. The bottom-up GGM algorithm evaluates the triple genes by comparing the difference between the correlation coefficient of the two bottom-layered genes and the partial correlation coefficient of the two bottom-layered genes given a TF at the immediately upper layer. When the difference is statistically significant, the TF was defined to be the regulator of the two bottom-layered genes [7]. This is because the correlation coefficient of the two bottom-layered genes represents their coordination in the presence of the TF, whereas the partial correlation coefficient reflects their coordination after the effect of the TF on both bottom-layered genes is removed. The difference represents the interference strength of the TF on two bottom-layered genes. Though bottom-up GGM is very efficient in identifying regulatory genes (nodes) associated with a pathway through constructing multilayered hGRNs, a large number of edges are generated in the resulting hGRNs owing to exhaustive combinations of all remained TFs in the pool and all paired bottom-layered genes [7]. Too many edges benefit novel TF recognition, but make it hard to identify the regulatory edges for conducting experimental validation. In addition, the regulatory strength of each edge in a network built with bottom-up GGM algorithm cannot be well characterized due to different regulation strengths among different triplet genes. For these reasons, we developed a backward elimination random forest (BWERF) for constructing MH-hGRNs, which can be used as an alternative approach especially when recognition of implicit regulatory edges is prioritized to be a goal. Compared to the bottom-up GGM algorithm, BWERF can significantly reduce the number of edges in the ML-hGRNs constructed. For example, the ML-hGRN operating above the ligncellulosic pathway constructed with BWERF had only 90 edges (Fig 3) in contrast to the 1507 edges in the ML-hGRN constructed with bottom-up GGM [7].

BWERF is based on the random forest model, and thus inherits the advantages of the random forest model in determining the important regulatory variables to each pathway gene. Firstly, the random forest model is applicable to the data sets where the number of variables is much larger than the number of samples. The random forest model uses randomly selected subset of variables at each splitting node, and thus important variables can be correctly separated from a large number of non-important regulatory variables. Secondly, the random forest model can detect non-linear regulatory relationships among variables. Thirdly, if only the parameter of *ntree* in random forest model is big enough, the random forest model will not be overfitted. Lastly, the criteria for choosing splitting variable in decision tree for the random forest model is to maximally decrease the variance of dependent variables, and the decreased variances are used to characterize the importance of the variables. Therefore, the variable importance values returned by the random forest model are more intuitive. BWERF inherits all these features of the random forest model that are critically important for reverse-engineering of gene networks from the data where gene variables far exceed the number of samples.

We used GENIE3 as a comparison method because it also uses the random forest model to construct gene regulatory networks. However, GENIE3 is not tailored for constructing ML-hGRNs. GENIE3 creates the random forest model for each pathway gene only once while BWERF creates the random forest model for each pathway gene multiple times as it conducts backward elimination in a recursive manner and BWERF also considers the high correlation among a group of pathway genes and uses it by aggregating the importance values of TFs for ranking TFs. As we know that the gene regulatory relationships are intricate and the number of TFs is much larger than the number of samples. Non-regulatory TFs to the pathway of interest can severely twist the importance values of regulatory TFs if they are not appropriately modeled. The backward elimination step has its value in two aspects: first, it helps the regulatory genes with medium-level regulatory strength to emerge from noise variables, as shown with the “toys data”; second, the backward elimination can enlarged the range of importance values of the variables, which makes true regulatory variables more differentiable from noise variables. Although it takes more computational time to run the backward elimination, we found that the running time is acceptable with moderate computational power. For example, when we used our Linux server with 22 cores to construct a ML-hGNR from a compendium *Arabidopsis thaliana* microarray data set with 128 samples, it took about one and half hours to run.

Compared to GENIE3, the best performer in DREAM4 *In Silico Multifactorial* challenge [24], BWERF obviously has increased accuracy owing to the backward elimination to exclude non-regulatory TFs. The backward elimination was conducted recursively to the input regulatory genes towards obtaining the genuine regulators peculiar to a pathway gene, each time with the controlled deletion rate to avoid overdoing, and to ensure only less important TFs were removed. In this paper, we used an elimination rate of 10% and found a considerable improvement and acceptable computational time with the use of this elimination rate. In fact, there was a noticeable improvement of BWERF over GENIE3 when the elimination rate was as high as 50%. When elimination rate was decreased from 50%, the order of the importance of TFs to a pathway gene changed slightly, but the variance of the importance of each TF decreased accordingly, resulting in a more stable importance list. Though the computing time increased accordingly, the benefit is overweighed the time cost, especially considering the years may be taken for biologists to conduct experimental validation To show the effect of different elimination rates on the “toys data set”, we enclosed the output in S3 File.

We certainly compliment GENIE3 for its great value in constructing well-stretched and well-connected “single-layered” GRN comprising of large number of genes. For constructing a multiple-layered hGRN operating over a given pathway, BWERF is tuned to have a higher accuracy and reliability for gene selection at low-level layer. Small derivation at the lower-level layered gene selection can lead to significant discrepancies at the high–level layers during construction of ML-hGRN. We will anticipate the experimental validation of our method in the next decade though we have begun to receive some positive feedback, for example, as what have been shown in our recent publication[4]. We believe the BWERF has its great value in recognition of authentic TFs and regulatory relationships in a ML-hGRN that governs a biological pathway or a process.

## Conclusions

A random forest algorithm with backward elimination was developed for constructing a ML-hGRN that operates above a given metabolic or canonical pathway using microarray or RNA-seq data sets. The algorithm was evaluated with both a synthetic “toys data set” and two real gene expression data sets from *Arabidopsis thaliana* and *Mus musculus* (mouse), leading to the networks with significantly enriched known positive regulatory genes and much less number of regulatory edges. The efficiency and accuracy were corroborated by both PR and ROC curves and the capture of positive regulators. Our method is especially useful for biologists who would like to identify the hierarchical regulators associated with a metabolic pathway or a biological process of interest from exploded gene expression data in public repositories.

## Materials and methods

### *Arabidopsis* microarray data sets

The *Arabidopsis* gene expression data used in this study were downloaded from public repository. The wood formation compendium data set contains the 128 microarrays pooled from six experiments, which have the accession identifiers of GSE607, GSE6153, GSE18985, GSE2000, GSE24781, and GSE5633, in NCBI Gene Expression Omnibus (GEO) (http://www.ncbi.nlm.nih.gov/geo/). These data sets were obtained from hypocotyledonous stems under short-day that is known to induce secondary wood formation[25]. All data sets mentioned above were derived from hybridization of Affymetrix 25 k ATH1 microarrays. The original CEL files were downloaded and processed by the robust multiarray analysis (RMA) algorithm using the Bioconductor package. For the quality control we used the methods that were previously described [26].

### Mouse microarray data sets

The mouse microarray data sets were download from Embryonic Stem Cells Atlas of Pluripotency Evidence (ESCAPE) website (http://www.maayanlab.net/ESCAPE/). We downloaded a time course gene expression data set from R1 ESCs under undirected differentiation using Affymatrix MOE430A array. The time-course include 0 h, 6 h, 12 h, 18 h, 24 h, 36 h, 48 h, 4 d, 7d, 9d, and 14 d, with 3 replicates at each time point. The data file downloaded was named as R1_ES_EB_MOE430A.txt.zip. The positive regulatory relationships were obtained from a file called chip_x.txt.zip, which contains the protein/DNA interactions table extracted from ChIP-X studies. ESCAPE includes 206,521 protein-DNA binding interactions in proximity to coding regions covering 48 ESC-relevant transcription factors and chromatin modifiers. The pathway selected was mouse pluripotency maintenance pathway with 24 genes (S1 File), which are regulated by 35 known TFs. Three datasets were generated by adding the profiles of 100, 200, and 300 randomly selected noise genes from the expression dataset into the profiles of 35 TFs.

### Construction of ML-hGRN with backward elimination random forest

In order to construct ML-hGRN that govern a biological pathway, the algorithm first placed pathway genes at the bottom (first) layer, and tried to identify the most significant regulatory genes (TFs) that are associated with the pathway genes with causal relationships, and then built the second layer of network. After that, the regulatory genes shown at the second layer were removed from the pool of input genes, and then used as the new bottom layer, and the remaining input genes were used as an input to construct the third layer. This process was repeated until the designated number of layers is achieved or no more layer can be built owing to lack of TFs that have causal relationships with the current bottom layer.

**Fig 4:**
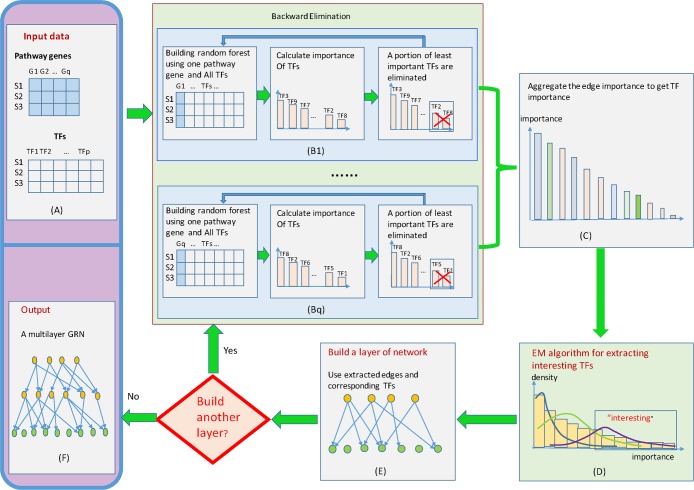
Flowchart illustrating BWERF algorithm for constructing multilayered hierarchical gene regulatory network using expression data of pathway genes and regulatory genes. A. Input for BWERF included a pathway gene expression matrix and a TFs expression matrix. B. For each pathway gene, recursively constructing of random forest model with backward elimination. C. Aggregation of the importance values of a TF to all pathway genes to produce a unified the importance value of this TF to the pathway. D. The Expectation-maximization (EM) algorithm was implemented to fit a Gaussian mixture model to the importance values. E. The most important TFs were identified and used as a layer. F. By using the new TF layer as bottom layer, we repeated all above procedure to obtain the next layer until the designated number of layer was achieved or the program was terminated due to the lack of significant TFs as input for upper layers.

Assume we have *p* TFs, *q* pathway genes and *n* samples, then the inputs are two expression matrices: TFs matrix with size *n* × *p* and pathway genes matrix with size *n* × *q* (Fig 4A). In order to construct one layer of GRN, we divided the inferring GRN problem into *q* different regression problems. For each regression problem, we took one pathway gene as response variable, took all TFs as predictor variables. To identify the significant regulatory relationships from TFs to a pathway gene is to ascertain the most important predictor variables in the regression problem. For each regression, we used the random forest method, which will be explained below, to identify the candidate regulatory relationships. We fitted the input data to a random forest model, recorded the importance value for each TF calculated from the model. Then, we sorted the TFs according to their importance values in decreasing order and eliminated certain portion (e.g. 1/10 or 1/5) of the least important TFs, we re-fit the remaining genes to the random forest model. This process was repeated until one TF is left (Fig 4B). The purpose of using such a backward elimination strategy was to reduce the chance for mistakenly deleting important TF genes. After every pathway gene was evaluated using above random forest model with backward elimination procedure, we aggregated all importance values of a TF to all the pathway genes to obtain a unified importance value for each TF (Fig 4C). Here, we allow users to add a weight to each pathway gene based on existing knowledgebase so that the regulatory relationships eventually identified will favor to some particular pathway genes. If no weights are available for pathway genes, users can enter 1 for each pathway gene. In order to identify one layer of gene regulatory network, users can assign the number of TFs empirically, or use expectation-maximization (EM) algorithm to fit a Gaussian mixture model to the importance values obtained, and extracted the most important TFs (Fig 4D). The TFs within the frame of interest as illustrated in Fig 4D have the highest probability belonging to the rightmost component, and were extracted. An edge will be added to each extracted TF and the linked pathway gene to form one-layered GRN (as shown in Fig 4E). By using the newly obtained TF layer as bottom layer, we repeated all above procedure to obtain the next upper layer until the designated number of layers was achieved or the program was automatically terminated due to the lack of significant TFs as input for upper layers.

### Details of the random forest model

Random forest is a machine learning technique developed by Leo Breiman [21]. It can be used on both classification and regression problems. In general, random forest uses bootstrap to generate a random subset of samples (data sets) from original data set, and then constructs an individual decision tree. For the root node, a subset of randomly selected variables (genes) were used as the candidates for partition of the aforementioned random subset of samples. The best candidate gene being selected for the root node is the one by which we can split the random subset of samples into two children subsets with the minimal impurity. The impurity is a measure of whether the similar response values end up in the same set (child) in a partition. For classification problem, it is typically either Gini impurity or information entropy; for regression problem it is variance. After the root was set, the aforementioned random subset of samples was partitioned into two children nodes. The decision tree was then built recursively in the same fashion as described above for each child node until the impurity for each leaf node is zero. When completed, the decision tree is called a fully grown tree. In this study, we used bootstrap to generate n (where n = 1000) random subsets of samples, and built 1000 decision trees, which were subsequently combined and used to evaluate all TFs in these trees.

To rank TFs associated with the genes in specific pathway, we need to learn the importance value of each TF to the pathway genes. Random forest can return the importance value of each independent variable in a natural manner. For a node of decision tree, the importance of the splitting variable is defined as the value of decreased variance using formula: *IV* = *nδ*_*p*_ − *n*_1_*δ*_*c*1_ − *n*_2_*δ*_*c*2_ where *p* stands for the parent node while c stands for the children, *δ*_*p*_ represents the variance of all *n* samples split at parent node while *δ*_*c*1_ and *δ*_*c*2_ represents the variance of all *n*_1_ samples split at children node C1 and variance of all *n*_2_ samples split at child node C2 respectively. The importance of other variables is defined as zero for this node. For a decision tree, the importance of each independent variable is the sum the importance in all nodes of the tree. For a random forest, the importance of each independent variable is the average of importance of the variable in all trees.

### Details of Expectation Maximization (EM) algorithm

Suppose *x*_1_,*x*_2_,…,*x*_*N*_ are the importance values of TFs that were obtained from BWERF. We assumed they are independent and identically distributed (i.i.d.) samples from a finite mixture of K > 1 Gaussian distributions. The density of each *x*_*i*_ can be written as

$$g(x_{i})=\sum_{k=1}^{K}\alpha_{k}\varphi_{k}(x_{i};\mu_{k},\sigma_{k}^{2})$$

where $\alpha_{k}>0,\;\sum_{k=1}^{K}\alpha_{k}=1$, *φ*_*k*_ is Gaussian density function. Let

$$\Theta =(\alpha_{1},\ldots ,\alpha_{K},\mu_{1},\ldots ,\mu_{K},\sigma_{1}^{2},\ldots ,\sigma_{K}^{2})$$

be the parameter vector want to be estimated. The EM algorithm is an iterative algorithm that starts from some initial estimate of Θ, and then proceeds to iteratively update Θ until convergence is detected. Each iteration consists of an E-step and an M-step.

**E-Step**: Denote the current parameter values as Θ. Compute the membership weight of *x*_*i*_ belongs to component k.

$$w_{ik}=p(x_{i}\;belongs\;to\;component\;k)=\frac{\alpha_{k}\varphi_{k}(x_{i};\mu_{k},\sigma_{k}^{2})}{\sum_{k=1}^{K}\alpha_{k}\varphi_{k}(x_{i};\mu_{k},\sigma_{k}^{2})}$$

for all data points *x*_*i*_, 1 ≤ i ≤ N and all mixture components 1 ≤ k ≤ K. This yields an N × K matrix of membership weights, where each of the rows sum to 1.

**M-Step**: Now use the membership weights and the data to calculate new parameter values. Let $N_{k}=\sum_{i=1}^{N}w_{ik}$, i.e., the sum of the membership weights for the k^th^ component. Then,

$$\alpha_{k}^{new}=\frac{N_{k}}{N},\;k=1,\ldots ,K$$

$$\mu_{k}^{new}=\frac{\sum_{i=1}^{N}w_{ik}x_{i}}{N_{k}},\;k=1,\ldots ,K$$

$$\sigma_{k}^{2\;new}=\frac{\sum_{i=1}^{N}w_{ik}{\left(x_{i}-\mu_{k}^{new}\right)}^{2}}{N_{k}}$$

When the parameters were obtained, the TFs that had the highest probability belonging to the component that had the largest mean in the Gaussian mixture model were identified to be the putative regulatory TFs.

### Comparison between BWERF and GENIE3

We compared BWERF with GENIE3, the latter is the best performer in DREAM4 *In Silico Multifactorial* challenge [24]. Huynh-Thu et al used random forest in GENIE3 to construct gene regulatory network. However, GENIE3 is not tailored to construct GRNs that govern biological pathways or processes. A network constructed with GENIE3 is more like a net with all directive connections but is lack of hierarchy. In order to use GENIE3 as a comparison, we enforced some additional rules to use the top TFs ranked by GENIE3 as a layer over the bottom layer of pathway genes. After that, a new layer was derived in the same principle. The number of TFs kept at each layer was the same as BWERF for the purpose of comparison with BWERF.

### Testing efficiency of BWERF and GENIE3

In order to evaluate and compare the efficiency of BWERF and GENIE3, we plotted the precision recall (PR) curve and receiver operating characteristic (ROC) curves for the results of two algorithms, and calculated the statistics AUPR and AUROC. The PR curve is created by plotting the precision against the recall at various threshold settings, while the ROC curve is created by plotting the true positive rate (TPR) versus the false positive rate (FPR). The definitions of precision, recall, TPR and FPR are as follows:

$$precision=\frac{TP}{TP+FP}$$

$$recall=TPR=\frac{TP}{P\_total}$$

$$FPR=\frac{FP}{F\_total}$$

Where TP, FP are the numbers of regulatory relationships above the threshold being true positive and false positive respectively. P_total and F_total are the total number of positive and negative regulatory relationships respectively. AUPR is calculated by Trapz [27] numerical integral of precision with respect to recall. AUROC is calculated by Trapz numerical integral of TPR with respect to FPR.

## Supporting Information

S1 File The mouse data and the edge list identified by BWERF. (ZIP)

S2 File The Arabidopsis data, the edge list identified by BWERF and Mapping between gene ID and gene symbol. (ZIP)

S3 File The effects of elimination rate on toys data. (DOCX)

S4 File The BWERF programme. (ZIP)
