# Supplementary material for: Recursive random forest algorithm for constructing multilayered hierarchical gene regulatory networks that govern biological pathways
Source: PLoS One. 2017 Feb 3;12(2):e0171532. doi: 10.1371/journal.pone.0171532 (PMC5291523; doi:10.1371/journal.pone.0171532)
Supplement: S3 File — (DOCX) [file pone.0171532.s003.docx]

**Fig. Effect of different elimination rate on “toys data”.**

When elimination rate is 50%, the importance values of true regulatory variables $x_{5}, x_{6}, x_{4}, x_{1}$ surpass the importance of noise variables. It is easier to differentiate the true regulatory variables from noise when the elimination rate is smaller. However, the computing time increase accordingly. The computing time was 121s for GENIE3. With the elimination rate of 50%, BWERF used 260s; with the elimination rate of 20%, BWERF used 639s; with the elimination rate of 10%, BWERF used 1157s. Although BWERF took longer when the smaller elimination rates were used, the running time for BWERF with the elimination rate of 10% was still acceptable and was much shorter that the time needed for validations by biologists. Therefore, we suggest choosing a smaller elimination rate.
